# Effect of Cold Rolling on the Evolution of Shear Bands and Nanoindentation Hardness in Zr_41.2_Ti_13.8_Cu_12.5_Ni_10_Be_22.5_ Bulk Metallic Glass

**DOI:** 10.3390/nano11071670

**Published:** 2021-06-25

**Authors:** Abhilash Gunti, Parijat Pallab Jana, Min-Ha Lee, Jayanta Das

**Affiliations:** 1 Department of Metallurgical and Materials Engineering, Indian Institute of Technology Kharagpur, West Bengal 721302, India; abhilash@iitkgp.ac.in (A.G.); parijat.pallab@iitkgp.ac.in (P.P.J.); 2 KITECH North America, Korea Institute of Industrial Technology, San Jose, CA 95134, USA; mhlee1@kitech.re.kr

**Keywords:** bulk metallic glass, Vitreloy 1, nanoindentation, cold rolling, densification, inhomogeneity

## Abstract

The effect of cold rolling on the evolution of hardness (*H*) and Young’s modulus (*E*) on the rolling-width (RW), normal-rolling (NR), and normal-width (NW) planes in Zr_41.2_Ti_13.8_Cu_12.5_Ni_10_Be_22.5_ (Vitreloy 1) bulk metallic glass (BMG) was investigated systematically using nanoindentation at peak loads in the range of 50 mN–500 mN. The hardness at specimen surface varied with cold rolling percentage (%) and the variation is similar on RW and NR planes at all the different peak loads, whereas the same is insignificant for the core region of the specimen on the NW plane. Three-dimensional (3D) optical surface profilometry studies on the NR plane suggest that the shear band spacing decreases and shear band offset height increases with the increase of cold rolling extent. Meanwhile, the number of the pop-in events during loading for all the planes reduces with the increase of cold rolling extent pointing to more homogeneous deformation upon rolling. Calorimetric studies were performed to correlate the net free volume content and hardness in the differently cold rolled specimens.

## 1. Introduction

Bulk metallic glasses (BMGs) exhibiting high structural strength can be synthesized by quenching from the liquid state at a critical cooling rate of >10^2^ K/s [1]. The glassy structure produced at different cooling rates exhibits different free volume contents, leading to the evolution of different configurational states in the potential energy landscape (PEL), and exhibits different fictive temperatures *T*_f_, which affects the mechanical behavior [2,3]. Owing to the absence of long-range order, BMGs are isotropic, but can become anisotropic in terms of elastic, mechanical, and magnetic properties owing to local cooling rate difference fluctuations during vitrification [4] or secondary processing like elastic [5], anelastic [6], homogeneous (creep) [7], and inhomogeneous plastic deformation through compression [8] or high-pressure torsion [9]. Bond-orientation anisotropy (BOA) in BMGs was reported to cause anelastic deformation [8,10]. Furthermore, local bond exchange or the bond reformation during plastic deformation was reported to be the root cause of the observed structural anisotropy [3,7,9].

The plastic deformation of bulk metallic glasses (BMGs) involves two competing processes, including free volume creation and relaxation [11,12,13]. The free volume creation process includes disordering and dilatation, which led to softening, whereas the relaxation process is linked with diffusional ordering and densification [14,15]. The disordering and softening processes dominate in most of the cases and fail the BMG catastrophically by forming shear bands [11,12,16]. At low temperatures, the run-away of shear bands is dominated by the sluggish diffusion rate, thus the free volume accumulation is significant compared with that of free volume annihilation rate. Therefore, experimental and molecular dynamics simulations have shown that uncontrolled softening is the dominant mechanism during shear banding, as reported by several authors [17,18,19,20,21,22,23,24,25,26].

On the other hand, the hardening in BMGs has been reported to occur as a result of the homogeneous nucleation and continuous multiplication of the shear bands [27]. Similarly, the change in the morphology and distribution of the chemically heterogeneous domains in the glassy structure may also contribute to the hardening [28]. Along this line, Lee et al. have reported an increase in the compressive yield strength in Vitreloy 1 upon cold rolling without any change in the local chemical composition nor the deformation induced nanocrystallization [29]. Interestingly, the strain-hardening has also been reported during cyclic compressive loading [30]. Schuh et al. have reported that the hardening in a glassy phase is linked with the formation of new shear bands, which increases the flow stress owing to the deformation induced nanocrystallization in the shear bands [13]. Recently, Wang et al. have also observed the densification in a monolithic BMG upon tensile deformation. Such densification is linked to the diffusional rearrangements leading to rapid annihilation of the free volume, which dominates over the shear flow and the free volume accumulation [31]. Therefore, in-depth studies are required to understand the deformation-induced relaxation and dilatation in the BMGs.

In the present work, monolithic Zr_41.2_Ti_13.8_Cu_12.5_Ni_10_Be_22.5_ (Vitreloy 1) BMG was cold rolled (CR) to different reductions and the variations of the nanoindentation hardness and Young’s modulus on the rolling-width (RW), normal-rolling (NR), and normal-width (NW) planes at different loads were systematically studied. The evolution of the shear bands on the NR plane was studied by optical profilometry. The effect of rolling on the hardness was correlated with the free volume content.

## 2. Experimental Procedure

**Figure 1:**
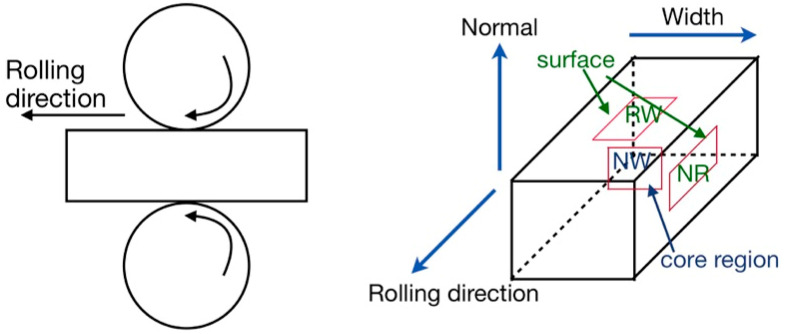
Schematic illustration of cold rolling and regions of normal-rolling (NR), rolling-width (RW), and normal-width (NW) planes for nanoindentation measurements.

**Figure 2:**
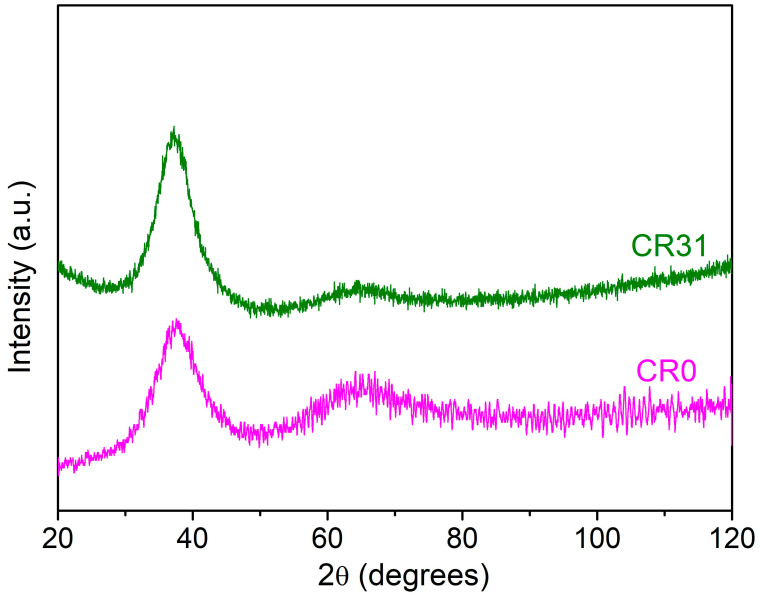
X-ray diffraction (XRD) patterns of CR0 and CR31 showing amorphous humps confirming the glassy nature of the samples.

Rectangular bars of Zr_41.2_Ti_13.8_Cu_12.5_Ni_10_Be_22.5_ (Vitreloy 1) BMG with the size of 2 × 2 × 4 mm^3^ were polished carefully to achieve a mirror-like surface finish and were cold-rolled (CR) up to 4.5% (CR4.5), 10% (CR10), 20% (CR20), and 31% (CR31), with 0.05 mm reduction during each pass, as shown in Figure 1. The structure of the specimens was characterized using a differential scanning calorimeter (DSC, Perkin Elmer, Waltham, MA, USA, DSC 8000) and X-ray diffraction (XRD), confirming the glassy nature of the as-cast (CR0) and the differently CR specimens. The X-ray diffraction studies were performed using Philips PANalytical X-ray diffraction (XRD) unit (PW3373, The Netherlands) with Cu-Kα radiation. Figure 2 displays the X-ray diffraction (XRD) patterns of CR0 and CR31, confirming the structure of the specimens to be amorphous. DSC experiments were carried out at a heating rate of 20 K/s. As the shear bands are visible on the free surfaces only, the NR plane of the CR specimens was investigated, which remained untouched during rolling to construct the 3D contour of the surface. The 3D contour of the surfaces was constructed using the interference pattern, as constructed by the 1376 pixel × 1032 pixel high resolution colour charge-coupled device camera attached to Bruker Contour GT 3D-optical surface profilometer (OSP). The instrument has a wide dynamic range with lateral resolution of 0.3 μm and RMS of < 0.03 nm. The shear band spacing and the offset height were estimated using Vision64 analysis software (Billerica, MA, USA).

**Figure 3:**
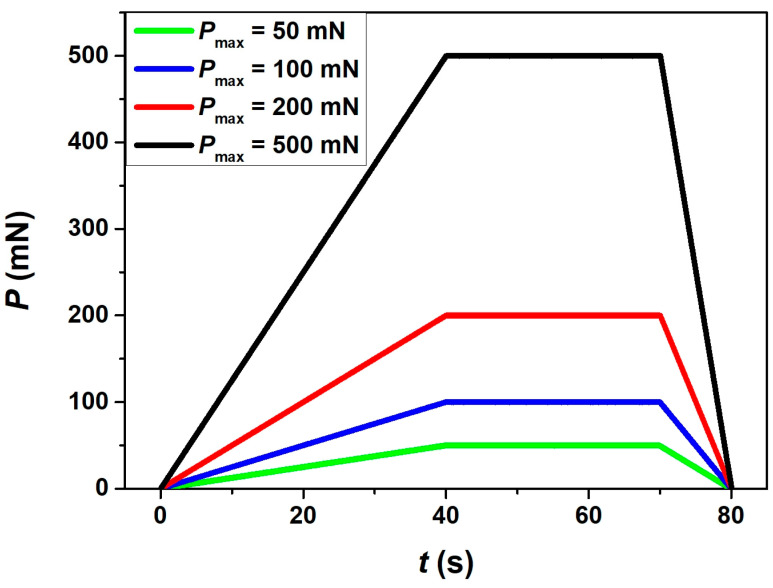
Schematic illustration of load (*p*)–time (*t*) plot used in the nanoindentation experiments.

Similarly, an optical microscope attached to the nanoindenter was used to choose the flat surface in between the two nearby shear bands for the indentation studies on the NR plane. A minimum distance was maintained between two indentations following ASTM standard, which is 20 times of the indentation depth, to avoid an overlapping of the two nearby plastically deformed zones. As no shear bands were observed on the RW and NW planes, the location for nanoindentation tests was chosen randomly, whereas the as-cast and CR samples were cut into two halves along the NW plane for the nanoindentation studies for NW planes, as depicted in Figure 1. The nanoindentation tests were performed using Hysitron TI950 TriboIndenter, Minneapolis, MN with a standard three-sided Berkovich indenter at room temperature in control loading rate (CLR) mode with varying maximum applied load (P_max_) at 50 mN, 100 mN, 200 mN, and 500 mN. Instrumented nanoindentation carried out by pressing a Berkovich indenter into the sample surface with of 30 s of loading followed by load holding of 40 s at peak load and unloading of 10 s, as illustrated in Figure 3. The nanoindenter load resolution is ±1 nN and displacement resolution is ±0.02 nm. The thermal drift was set to be ±0.05 nm/s. The contact area between the diamond indenter and the specimen was calibrated using a fused-quartz standard [32]. A field emission scanning electron microscope (FESEM, Zeiss Merlin Gemini 2, Zeiss, Jena, Germany) was used to study the nanoindentation impressions in the as-cast and differently CR samples. The hardness (*H*) and elastic modulus (*E*) values were calculated from *P*-*h* curves using the Oliver and Pharr model [32].

## 3. Results

### 3.1. DSC Studies and Free Volume Estimation

**Figure 4:**
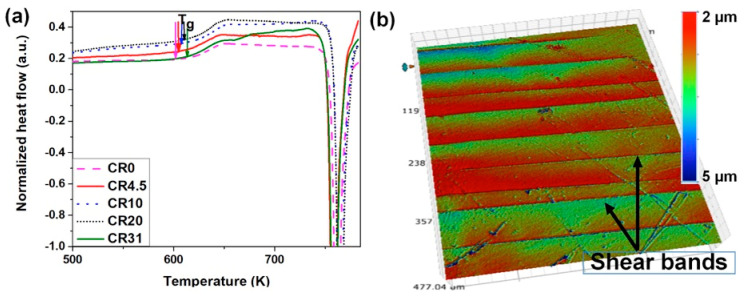
(**a**) Differential scanning calorimeter (DSC) plots of CR0, CR4.5, CR10, CR20, and CR31 showing the glass transition event followed by crystallization. (**b**) 3D optical profile showing the evolution of shear bands with step-like features on the surface of CR10.

Figure 4a displays the DSC traces for CR0, CR4.5, CR10, CR20, and CR31. The onset of glass transition ($T_{g}^{onset}$) was estimated to be 609.8 ± 8.0 K (CR0), 611 ± 3.7 K (CR4.5), 616.6 ± 7.5 K (CR10), 619.1 ± 3.8 K (CR20), and 619.9 ± 5.5 K (CR31). The estimated $T_{g}^{onset}$ values of CR specimens are higher than that of CR0. The onset of the crystallization (*T*_x_) was measured to be 752.5 ± 0.1K (CR0), 759.9 ± 0.1 K (CR4.5), 760.1 ± 0.2 K (CR10), 759.2 ± 0.1 K (CR20), and 756.7 ± 0.1 K (CR31), respectively, whereas the enthalpy change (Δ*H*) during crystallization was estimated to be −78.78 ± 0.46 J/g (CR0), −78.53 ± 0.09 J/g (CR4.5), −78.6 ± 0.33 J/g (CR10), −77.93 ± 0.63 J/g (CR20), and −81.08 ± 0.57 J/g (CR31), pointing to similar Δ*H* values of the as-cast and CR specimens. Hence, the glassy structure remains similar upon cold rolling without any hint of nanocrystallization. Such a conclusion was also confirmed by transmission electron microscopic investigation, and crystallization did not occur upon cold rolling [33]. Several researchers have shown an increase of Δ*H* values with an increase in the extent of cold rolling [11,12,13,14,34]. In contrary, Flores et. al. have shown that Δ*H* values varied with prior deformation [35].

According to Beukel and Sietsma [16], the reduced free volume (*x*) is expressed as *x = v_f_/*(γv*), where the average free volume per atom is *v_f_*, v* represents the critical free volume for atomic diffusion, and the overlap factor is indicated by *γ.* For simplicity, the reduced free volume (*x*) is named as free volume from here onwards in this work. The *x* value varies with the temperature when a metallic glass is subjected to continuous heating during DSC experiments. The glassy structure relaxes and attains the equilibrium upon heating at a constant rate below *T*_g_ [16]. The stored enthalpy releases during the reduction in the free volume and, therefore, the structural relaxation process is featured by an exothermic event. On the other hand, the rise in *x* with temperature after relaxation leads to an increase in the enthalpy and was characterized by an endothermic reaction, which is related to the glass transition [34]. The onset of the endothermic reaction, pointing to the rise in *x*, is called the onset of glass transition temperature or $T_{g}^{onset}$. At $T_{g}^{onset}$, *x* reaches the equilibrium free volume *x_eq_* $\left(T_{g}^{onset}\right)$, which can be expressed as follows:

(1) $$x_{eq}\left(T\right)=\frac{\left(T-T_{o}\right)}{B}$$

where T is the temperature at which free volume is measured; *T_o_* is the Vogel–Fulcher–Tammann (VFT) temperature (i.e., 390 K); and *B* is the product of *T_o_* and the fragility parameter, reported to be 9282 [16,36].

A proportional relation exists between the Δ*H* and the free volume change (Δ*x*) during both the structural relaxation and the glass transition event [6,34]:

Δ*H* = *A* · Δ*x* (2)

where *A* is the proportionality constant. The structural relaxation process in BMGs occurs at temperatures in between room temperature and $T_{g}^{onset}$. The change in the enthalpy throughout the structural relaxation process is related to the variation of the net free volume Δ*x*, which is a measure of the difference between *x*(*T_RT_*) at 297 K and the free volume at $T_{g}^{onset}$ in Vitreloy 1. Thus, the value of *x*(*T_RT_*) was estimated to be 0.0234, 0.0222, 0.0245, 0.0241, and 0.0229 using Equations (1) and (2) for CR0, CR4.5, CR10, CR20, and CR31, respectively. The *x*(*T_RT_*) value of CR0 is similar to that reported by Masuhr et al. [37]. The value of *x*(*T_RT_*) of CR10 and CR20 increased, whereas the same value of CR4.5 and CR31 decreased compared with that of CR0. Both the increase and decrease of free volume content upon plastic deformation of BMGs were reported earlier [11,12,13,14,21,38,39].

### 3.2. Characterization of Shear Bands on the NR Plane

**Figure 5:**
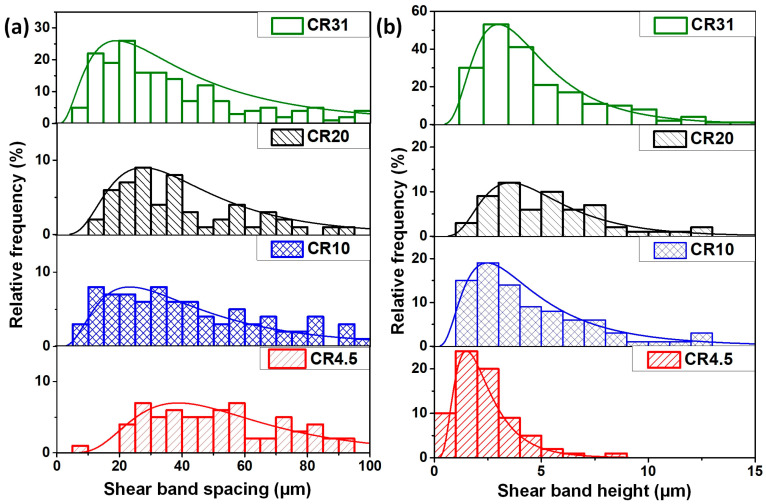
Histogram plot showing the distribution of (**a**) shear band spacing and (**b**) shear band offset height in CR specimens.

Three-dimensional (3D)-OSP studies were performed to quantitatively estimate the shear bands on the free surface of the NR plane, as the shear bands are visible on the free surface, which remained untouched during rolling. For example, a 3D-OSP image of shear bands formed on CR4.5 is shown in Figure 4b. The shear band spacing (λ) and the offset height (δ) were measured in each different CR sample by analyzing the 3D-OSP images, and their relative frequency distribution data shown in Figure 5a,b are very well fitted using the lognormal function, which has a regression coefficient of >0.9 in all cases. The shear band density increased with the increase of cold rolling percentage. It was observed that the λ value decreased and δ increased with the increase in the extent of cold rolling. The average value of shear-band spacing (λ_avg_) was estimated to be 37.6 ± 17.1 μm, 23.3 ± 19.7 μm, 26.8 ± 15.6 μm, and 19.3 ± 12.1 μm for CR4.5, CR10, CR20, and CR31, respectively, whereas the average value of shear band height (δ_avg_) was estimated to be 1.7 ± 1.2 μm, 2.6 ± 1.8 μm, 3.4 ± 0.9 μm, and 2.8 ± 2.1 μm for CR4.5, CR10, CR20, and CR31, respectively.

### 3.3. Nanoindentation Studies at Various P_max_ (50 mN–500 mN)

**Figure 6:**
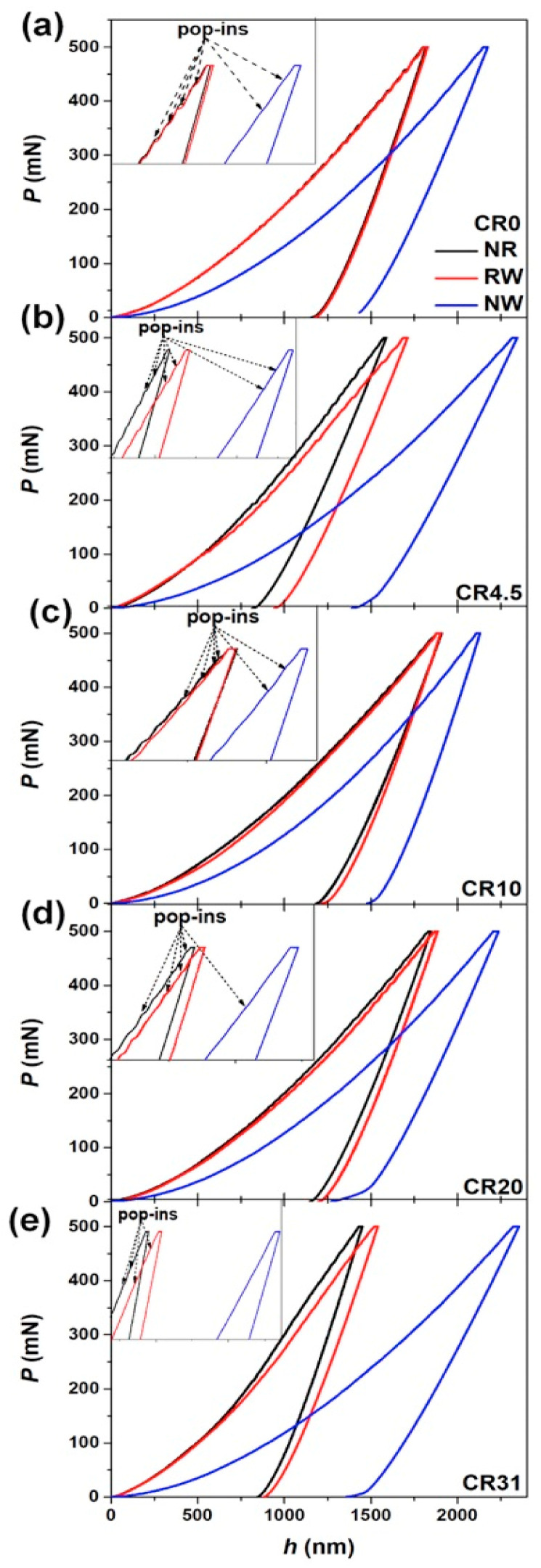
*P*-*h* plots at *P*_max_ = 500 mN for NR, RW, and NW planes: (**a**) CR0, (**b**) CR4.5, (**c**) CR10, (**d**) CR20, and (**e**) CR31; pop-in events are more pronounced in CR0, which gradually decreases with the increase of cold rolling percentage.

Figure 6a–e and Figures S1–S3 show the load (*P*) versus displacement (*h*) plots of CR0, CR4.5, CR10, CR20, and CR31 at different loading rates of 1.66, 3.33, 6.67, and 16.67 mN/s loaded up to *P*_max_ = 50 mN, 100 mN, 200 mN, and 500 mN, respectively, for NR, RW, and NW planes. The indentation hardness (*H*) was estimated using the following equation:

*H* = (*P*_max_/*A*_c_) (3)

where *A*_c_ is the corrected contact area, which is equal to C_1_*h*_c_^2^ + C_2_*h*_c_ + C_3_*h*_c_^1/2^ + C_4_*h*_c_^1/4^ + C_5_*h*_c_^1/8^ + C_6_*h*_c_^1/16^, and the C_n_ terms are constants, as described earlier [32,40]. The *h*_c_ values were corrected as *h*_c_ = *h*_max_ − 0.75(*P*_max_/*S*), where *S* is the unloading stiffness and *h*_max_ is the maximum penetration depth. The elastic modulus (*E*) of the specimen was measured using the following equation:

(4) $$\frac{1}{E_{r}}=\frac{1-\nu^{2}}{E}+\frac{1-\nu_{i}^{2}}{E_{i}}$$

where the reduced modulus $E_{r}=0.5\sqrt{\pi /A}\;\left(dp/dh\right)$, $E_{i}$ is the modulus of the indenter, and $\nu_{i}^{\;}$ is the Poisson’s ratio of the indenter. The Poisson’s ratio of Vitreloy 1 sample is *ν* = 0.37 [41].

**Figure 7:**
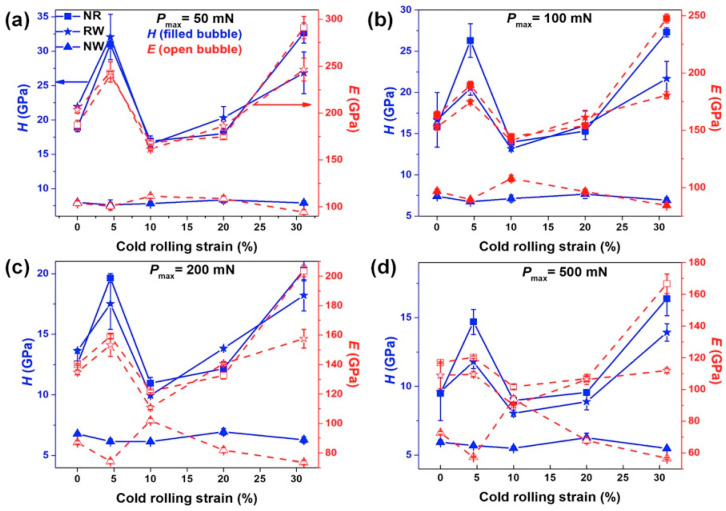
Hardness (*H*) and Young’s modulus (*E*) variation with cold rolling percentage in the NR, RW, and NW planes at *P*_max_ of (**a**) 50 mN, (**b**) 100 mN, (**c**) 200 mN, and (**d**) 500 mN.

It was noticed that the *h*_max_ value for a given load is higher on the NW plane than that of the NR and RW planes. In addition, the *h*_max_ values are higher in CR10 and CR20, and lower in CR4.5 and CR31 for a given load than that of CR0 for the NR and RW planes, as shown in Figure 6a–e. Figure 7 displays the variation *H* and *E* with the cold rolling percentage for *P*_max_ of 50 mN, 100 mN, 200 mN, and 500 mN for the NR, RW, and NW planes. The estimated *H* values of CR0, CR4.5, CR10, CR20, and CR31 were 9.6 ± 0.1 GPa, 14.8 ± 0.3 GPa, 8.9 ± 0.2 GPa, 9.5 ± 0.3 GPa, and 15.9 ± 0.1 GPa, respectively, for the NR plane, whereas the *H* values of the RW plane were estimated to be 9.6 ± 2.1 GPa, 11.8 ± 0.5 GPa, 8.1 ± 0.2 GPa, 8.9 ± 0.6 GPa, and 13.9 ± 0.7 GPa for CR0, CR4.5, CR10, CR20, and CR31, respectively. Furthermore, the *H* values were estimated to be lower for the NW plane as 5.9 ± 0.2 GPa, 5.7 ± 0.1 GPa, 5.5 ± 0.1 GPa, 6.3 ± 0.3 GPa, and 5.5 ± 0.1 GPa in CR0, CR4.5, CR10, CR20, and CR31, respectively. The values of *H* and *E* in CR0, CR4.5, CR10, CR20, and CR31 at various *P*_max_ values in between 50 and 500 mN for the NR, RW, and NW planes are presented in Table 1, Table 2 and Table 3, respectively. Even though the hardness fluctuation on a particular plane at a given cold rolling percentage is small, as evident from the above hardness error values, the variation of *H* and *E* with cold rolling percentage on the NR and RW planes is significant and shows a similar trend at all *P*_max_ values, as shown in Figure 7a–d. The effect of cold rolling percentage on the *H* and *E* values is lesser on the NW plane than that of the NR and RW planes. Therefore, the glassy structure was modified upon cold rolling, leading to the variation of *H* and *E* values with the increase of cold rolling percentage in Vitreloy 1.

Figure 6 and Figures S1–S3 display the analogous nature of the *P-h* curves on the NR and RW planes, with more pop-in events in the case of CR0 owing to more trapped in free volume upon cooling than that of the interior of the NW plane, which exhibited a more relaxed glassy structure. Meanwhile, a more parabolic nature of *P-h* curves with fewer serrations was observed on the NW plane in CR0 with less pop-in depth, revealing a more homogeneous deformation in the NW plane than that of the NR and RW planes. The lower number of pop-in events during nanoindentation on the NW plane than on the NR and RW planes indicates a low number of shear band activation and their propagation. Such reduced pop-in events due to the cooling rate difference between the sample surfaces and interior portion of Zr-based BMG rods have been observed earlier [42]. Likewise, all the cold rolled samples have shown similar behavior on the different planes at a given cold rolling strain.

### 3.4. Studies on the Indentation Impression under SEM

**Figure 8:**
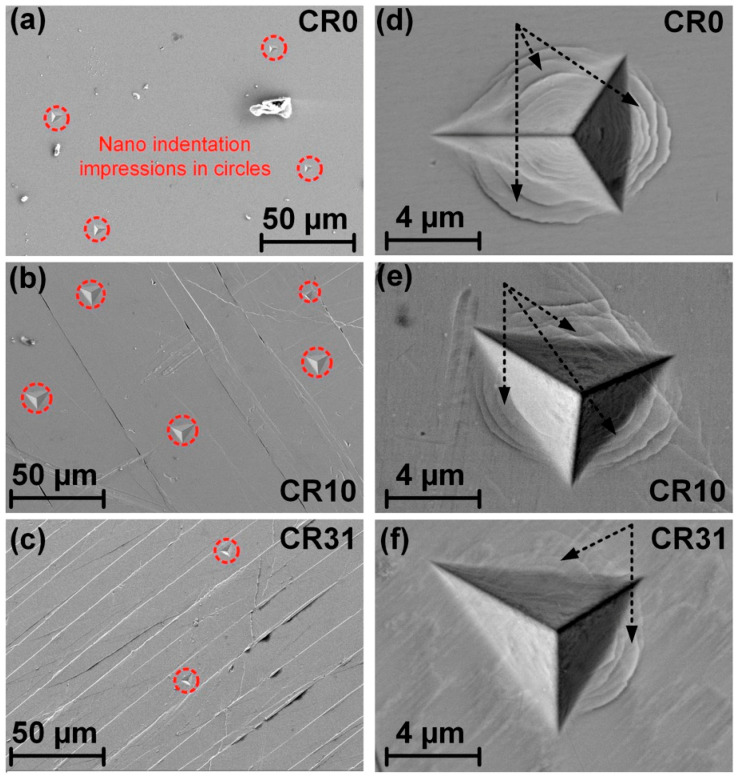
SEM secondary electron (SE) images showing nanoindentation marks in (**a**) CR0 along with evolved shear bands on the NR plane upon cold rolling in (**b**) CR10 and (**c**) CR31. The magnified image of indentation impression at *P*_max_ = 500 mN showing shear bands in (**d**) CR0, (**e**) CR10, and (**f**) CR31.

Figure 8a shows the indentation impressions, which are located 70–100 μm away from each other on the surface of CR0. The indentation impression under *P*_max_ = 500 mN in between two shear bands in the case of CR10 and CR31 is shown in Figure 8b,c, respectively. It is worth mentioning that *δ*_avg_ varied between 1.7 μm (CR4.5) and 2.8 μm (CR31) for different CR specimens, exhibiting a much larger length scale than that the indentation impressions for the NR plane. As the location for indentation was chosen between the flat region between two shear bands, the error on the measurement of *h*_c_ and subsequently on *H* and *E* must be neglected. Furthermore, Figure 8d shows the indentation impression on CR0 at *P*_max_ = 500 mN, which shows the formation of very finely spaced shear bands formed around as well as beneath the impression, suggesting inhomogeneous deformation. Meanwhile, a reduction in the number of shear bands around and beneath the indentation impressions was noticed, as shown in Figure 8e,f. Such a disappearance becomes more pronounced with the increase of cold rolling extent, implying the evolution of homogeneous deformation in cold rolled samples.

## 4. Discussion

### 4.1. Effect of Cold Rolling on Elastic-Plastic Response

**Figure 9:**
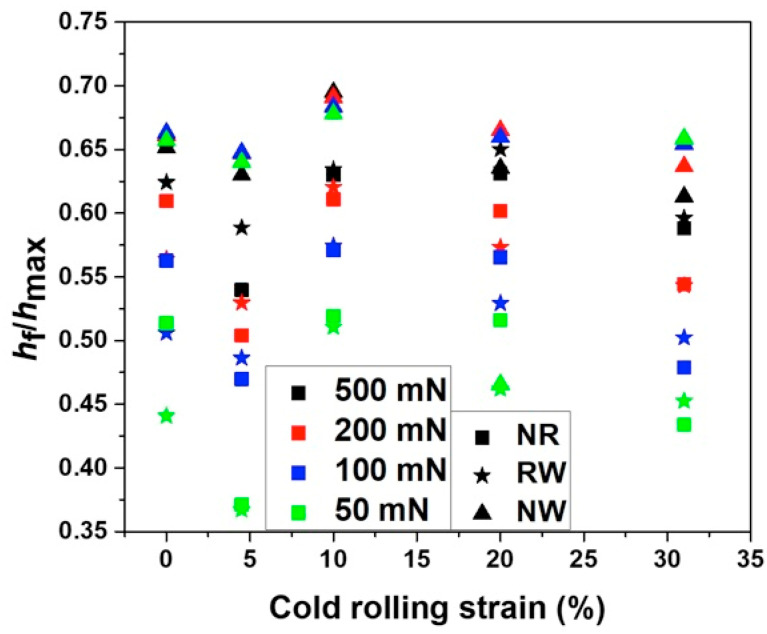
The *h*_f_/*h*_max_ ratio with cold rolling percentage for the NR, RW, and NW planes at *P*_max_ of 50 mN, 100 mN, 200 mN, and 500 mN.

The *P-h* plots of a specific sample for a given plane (either NR, RW, or NW) coincided with each other for all the *P*_max_ values in the range of 50–500 mN, which supports the repeatability and reproducibility of the experiments. The nature of elastic-plastic deformation beneath the indenter can be evaluated using the *h*_f_/*h*_max_ ratio, which lies between 0 and 1 [43]. *h*_f_/*h*_max_ = 0 implies a complete elastic deformation, whereas *h*_f_/*h*_max_ = 1 points to a fully plastic deformation. Figure 9 shows the *h*_f_/*h*_max_ ratio at different *P*_max_ of all the CR specimens for the NR, RW, and NW planes. The CR0 and different CR specimens showed *h*_f_/*h*_max_ < 0.7, indicating the deformation to be elastic-perfectly plastic. The effect of cold rolling percentage on *h*_f_/*h*_max_ exhibits a similar trend to that of *H* variation with cold rolling, as depicted in Figure 7a–d, whereas *h*_f_/*h*_max_ values lay between 0.6 and 0.7, depicting a more plastic nature in the NW plane than that for the NR and RW planes for all samples. Furthermore, more plastic behavior was observed in CR4.5 and CR31 than that of CR0, CR10, and CR20 for the RW and NR planes. Pharr et al. have shown, using experimental and finite element simulations, that pileup is not a significant factor when *h*_f_/*h*_max_˂ 0.7 and, hence, the Oliver–Pharr data analysis procedure can give reasonable results [32]. In the case of *h*_f_/*h*_max_ > 0.7, the pileup can result in wrong estimation of *H* owing to an erroneous contact area deduced from indentation load-displacement data [44,45]. In the present study, *h*_f_/*h*_max_ values < 0.7, indicating calculated H values, are realistic in nature for all samples for the NR, RW, and NW planes. 

**Figure 10:**
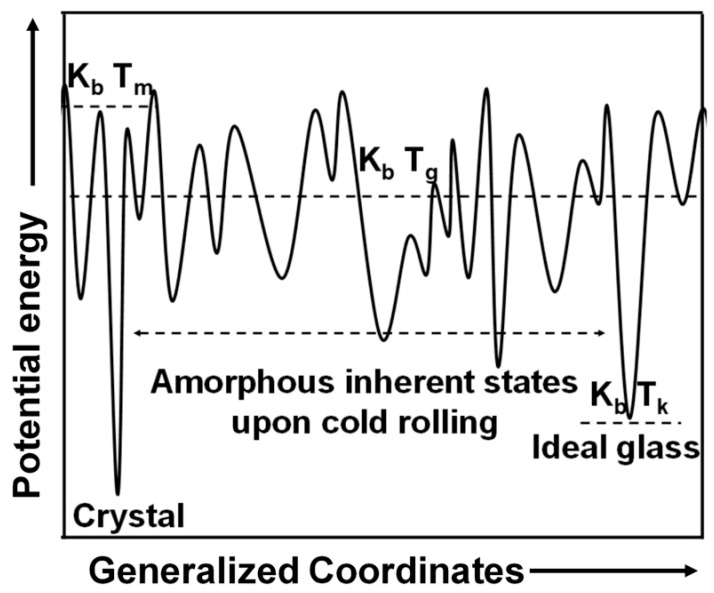
Schematic potential energy landscape (PEL) model illustrating different metastable energy states in the glassy phase.

The affected volume during nanoindentation was estimated by (3*h_f_)^3^ for all the specimens at all *P*_max_ for NR, RW, and NW, as shown in Table 4 [46]. The affected volume during nanoindentation is more than 0.4^3^ µm^3^, 0.7^3^ µm^3^, 1.3^3^ µm^3^, and 2.6^3^ µm^3^ for *P*_max_ of 50 mN, 100 mN, 200 mN, and 500 mN, respectively, for the NR and RW planes for all the samples. The hardened CR4.5 and CR31 show less affected volume during indentation than the CR0 and softened CR10 and CR20. The affected volume during nanoindentation in the central core of CR specimen on NW plane is estimated to be >1.2^3^ µm^3^ at all *P*_max_ values. Such an affected volume during nanoindentation validates a homogeneous deformation and corroborates the measured *H* and *E* values. A schematic potential energy landscape (PEL) model illustrates the available energy for glasses state: the lowest energy minima configuration for crystal representing corresponding atomic configurations or inherent states, as shown in Figure 10. No compositional variation was noticed on the three perpendicular planes of the specimens; therefore, the variations of *H* and *E* are linked to the evolution of different structural states only. Such structural fluctuations may evolve during synthesis owing to cooling rate differences between surface planes (NR and RW) and the interior core of the NW plane. NR and RW planes have shown similar *H* and *E* values, representing a similar glassy structure to that of the interior NW plane, which exhibited lower *H* and *E* values. Such an observation of different structural state formation was also reported in BMGs owing to cooling rate differences between the surface and core region of the sample [42]. Even though no compositional variation was observed in the specimens [33], the variation of *H* and *E* are linked to the different structural states that evolved upon cold rolling. Therefore, the evolution of structural fluctuations must have occurred with the increase of cold rolling strain on the NR and RW planes, which resulted in hardening in CR4.5 and CR31, whereas softening was observed in CR10 and CR31, as shown in Figure 4. Therefore, *H* and *E* values on the NW plane have shown only slight variation with increasing cold rolling strain indicating less fluctuations of the structural states.

### 4.2. Serrated to Smooth Flow on NR, RW, and NW Planes upon Cold Rolling

Strain bursts or pop-in events during loading portion of the *P*-*h* curve were observed owing to the nucleation of shear bands and/or their propagation, whereas elbow nature at the end part of the unloading *P*-*h* curve was also observed. The elbow-like step in the *P-h* curve is due to phase transition or softer regions or grain boundaries or pores in crystalline specimens. Such “pop-in” events were observed to be more in CR0 and a smoother flow curve was achieved in CR specimens, as depicted in Figure 6 at *P*_max_ = 500 mN for the NR, RW, and NW planes. A similar observation was made for all other *P*_max_ values, as depicted in Figures S1–S3. In addition, *P-h* curves exhibited more serration behavior for the NR and RW planes in CR0 and all CR samples than that for the NW plane at all *P*_max_ values.

The plastic event in a glassy phase is associated with the evolution of two different spatial regions, i.e., the shear transformation zones (STZs) and the residual glassy matrix [13]. The macroscopic plastic events proceed with the nucleation and proliferation of new shear bands. Such a phenomenon can be explained from the viewpoint of the percolation of the SRO and the atomic rearrangements in the glassy structure. If the local $\dot{\varepsilon }$ reaches a critical value, then the atomic rearrangements cannot further accommodate the applied strain. Hence, the differences of strain rates among the surrounding matrix and STZs would still remain, which results an inhomogeneous (non-Newtonian) flow and serrations during nanoindentation as reflected in the *P*–*h* curves, as shown for CR0 in Figure 6. The nature of the *P-h* curves for NR and RW planes have changed from ideal stair step-like behavior for CR0 and CR4.5 (lower cold rolling reductions) to a more parabolic nature with increasing cold rolling extent for all loading rates in the NR, RW, and NW planes, pointing to plastic deformation being more homogeneous upon cold rolling for all planes. Similar behavior of reduced pop-in events with a cooling rate difference between surfaces to the interior portion of the transverse direction was observed in Zr-based BMG rods [42]. Such features were supported by the disappearance of shear bands around the indentation impressions in CR31, as shown in Figure 8f. The reduction of the shear bands around the indentation impressions in CR samples also confirms the homogeneous deformation behavior upon cold rolling. Similarly, high-pressure torsion (HPT) processed BMGs also showed more homogeneous deformation [47].

### 4.3. Effect of Cold Rolling on Structure, H and E

The deformation of BMGs could be understood by the free volume theory as proposed by Spaepen, and Argon has suggested the STZ model involving clusters of atoms undergoing cooperative shear displacements [11,12,14]. The shear stress and indentation hardness are correlated as follows: $H\approx 3\sqrt{3}\tau$. According to the free volume model, the evolved shear strain rate ($\dot{\gamma }$) is influenced by the temperature (*T*), applied shear stress (τ), free volume accumulation, and viscosity (η), which is defined by the ratio of shear stress and shear strain rate as follows [11,14]:

(5) $$\dot{\gamma }=2c_{f}\alpha_{o}k_{f,O}\frac{\varepsilon_{o}\upsilon_{o}}{Ω}sinh\left(\frac{\tau \varepsilon_{o}\upsilon_{o}}{2k_{B}}\right)exp\left(\frac{-\Delta G}{k_{B}T}\right)$$

(6) $$\eta =\frac{\tau }{\dot{\gamma }}=\frac{\tau }{2fsinh\left(\tau \Omega /2k_{B}T\right)}\cdot exp\left(\frac{\chi v^{*}}{v_{f}}+\frac{\Delta G^{m}}{k_{B}T}\right)$$

(7) $$H=\frac{6\sqrt{3}k_{B}T}{\Omega }sinh^{-1}\left[\frac{\dot{\gamma }}{2f\cdot \gamma_{0}}\cdot exp\left(\frac{\chi v^{*}}{v_{f}}+\frac{\Delta G^{m}}{k_{B}T}\right)\right]$$

where Ω indicates the atomic volume, *α*_o_ is a coefficient featuring the quantity of the material undergoing shear transformation, $\varepsilon_{o}\upsilon_{o}$ denotes a flow event activation volume, τ is the shear stress, *k*_B_ is the Boltzmann constant, ΔG is the activation energy for defect migration, and *k*_f,o_ is associated with the Debye frequency.

The atomic radii of the elements Zr, Ti, Cu, Ni, and Be are 155 pm, 140 pm, 135 pm, 135 pm, and 112 pm, respectively, and the individual atomic volume of these elements was estimated to be 0.0156, 0.115, 0.0103, 0.0103, and 0.00588 nm^3^, respectively. Therefore, the average atomic volume (i.e., Voronoi volume) in Vitreloy 1 is 0.01165 nm^3^ [48]. The activation volume of the flow event ($\varepsilon_{o}\upsilon_{o}$) was estimated using nanoindentation data. The strain rate sensitivity (*m*) was estimated using the log(*σ*)–log($\dot{\varepsilon }$) plot, where $m=\frac{\mathrm{dln}\left(H\right)}{\mathrm{dln}\left(\dot{\varepsilon }\right)}$, activation volume $V=\frac{3\surd 3\left({\;k}_{B}\right)T}{mH}$, *H* is the indentation hardness at temperature *T*, and *k*_B_ is the Boltzmann constant. Assuming the tip radius of the Berkovich indenter is 25 nm, which remains constant for all experiments, $\tau_{max}$ was estimated as $\tau_{max}=\frac{0.47PR}{h}$. By considering ΔG, *α*_o_, *k*_B_, *T*, and *k*_f,o_ to be constant, the $\frac{\dot{\gamma }\;}{{\dot{\gamma }}_{0}\;}$ ratio was estimated to be 1, 0.93, 0.30, 0.71, and 0.61, for CR0, CR4.5, CR10, CR20, and CR31, respectively. The variation of $\dot{\gamma }$ indicates a change in the flow defect concentration, which will either increase or decrease the free volume content in order to accommodate the applied strain into the glassy phase. The application of shear stress during cold rolling alters PEL by reducing or eliminating the barrier to a neighboring/different metastable state [33]. Hence, the evolved $\dot{\gamma }$ in the CR specimens largely differs from that of as-cast BMG, further confirming the modification of the SRO cluster upon cold rolling.

As insignificant variation of *H* and *E* values with cold rolling percentage was noticed at the central core of the specimens on the NW plane, Spaepen’s free volume model was adopted to understand the observed behavior for the NR and RW planes. According to Spaepen’s model, the rate of free volume accumulation and annihilation can be estimated using the following equation [11].

(8) $${\left(\frac{dx}{dt}\right)}_{+}=\frac{f}{\gamma }exp\left(-\frac{\Delta G^{m}}{kT}\right)exp\left(-\frac{1}{x}\right)\left[cosh\left(\frac{\tau \Omega }{2kT}\right)-1\right]\frac{2kT}{xSv^{*}}$$

(9) $${\left(\frac{dx}{dt}\right)}_{-}=-fx^{2}\left[exp\left(-\frac{1}{x}\right)-exp\left(-\frac{1}{x_{eq}}\right)\right]exp\left(-\frac{\Delta G^{m}-\sigma_{m}V}{kT}\right)$$

where $S=\frac{2\mu \left(1+\vartheta \right)}{3\left(1-\vartheta \right)}$, µ is shear modulus, ϑ is Poisson’s ratio, ΔG is the activation energy, *f* is an attempt frequency, *k* is Boltzmann’s constant, Ω is atomic volume, $v^{*}$ is critical volume, γ is the geometrical factor, and the mean stress $\sigma_{m}$ = 0 during rolling operation. The values of the above parameters are *T* = 300 K, *γ* = 0.15, *f* = 5.415 × 10^12^ s^−1^, Ω = 2.424 × 10^−29^, µ = 35.3 GPa, v^*^ = 1.67 × 10^−29^m^3^, $\Delta G^{m}$ = 10^−19^ J, and ϑ *=* 0.37 [48]. The values of activation volume (*V*) and free volume (*x* and *x*_eq_) were obtained from the nanoindentation and DSC studies, respectively. Equation (8) indicates that the free volume accumulation depends on the applied shear stress, whereas Equation (9) indicates that free volume annihilation is linked with the diffusional atomic movement, which is further accelerated by the applied stress. Our calculation suggests that the free volume accumulation rate is 2.29 × 10^−19^ (CR0), 6.74 × 10^−20^ (CR4.5), 4.15 × 10^−6^ (CR10), 2.20 × 10^−14^ (CR20), and 6.42 × 10^−20^ (CR31). On the other hand, the free volume annihilation rates in CR0, CR4.5, CR10, CR20, and CR31 were estimated to be 5.09 × 10^−20^, 1.80 × 10^−15^, 5.56 × 10^−20^, 1.888 × 10^−18^, and 2.98 × 10^−19^, respectively. Plastic deformation in BMGs occurs inhomogeneously by forming shear bands at temperature <*T*_g_ and the coalescence of the free volume occurs gradually; therefore, the BMGs exhibit a dynamic response during nanoindentation.

Usually, the microhardness of BMGs decreases with prior deformation, as observed in Pd-base, Zr-base, and Cu-base glassy alloys [12,21,33,49]. Several authors have also reported an increase in hardness in BMGs upon deformation [13,28,29,30,31,32,34]. Furthermore, the increase of the yield strength of Vitreloy 1 upon cold rolling or laser shock peening was reported by Lee et al. and Cao et al., respectively [30,34,50]. The rise and fall of average free volume content with increasing cold rolling percentage were linked to the competition between the average free volume annihilation rate and creation rate, as studied using positron annihilation spectroscopy and DSC studies [33,35]. Furthermore, Pan and co-workers have proved that work hardening occurred as a result of densification in tensile pre-strained Zr-based BMGs using micro and nanoindentation, when free volume annihilation rate dominated the free creation rate [31,51].

**Figure 11:**
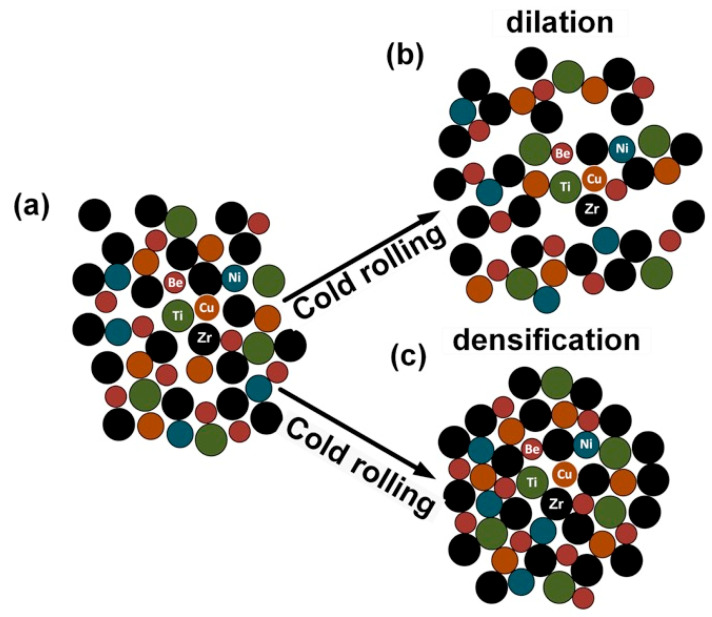
Schematic illustration of (**a**) the atomic arrangement in the glassy structure, (**b**) local dilation causing enhanced free volume content, as well as (**c**) densification during cold rolling.

Equations (5) and (6) indicate that the viscosity and indentation hardness decrease with the increase of free volume content. Turnbull and Cohen suggested that the excess free volume in MGs decreases the atomic bonding energy by increasing the average interatomic distances [52]. As the value of *E* is linked to the atomic bond strength, the increase of the *E* in CR4.5 and CR31 points to an increase of the bond strength compared with that of CR0. Meanwhile, the decrease of *E* in CR10 and CR20 points to a decrease in the bond strength compared with that of CR0 [53,54]. Thus, short range ordered (SRO) clusters in the glassy phase must have been modified into a newer atomic configuration metastable state upon cold rolling in the PEL model, as proposed by us [33]. In the case of CR10 and CR20, the decreas of the hardness is linked to a higher free volume content, as reported earlier [12,21,49]. Hence, the synergetic effect of free volume accumulation and annihilation during cold rolling is responsible for producing a relaxed glassy structure in CR4.5 and CR31 and a dilated glassy structure in CR10 and CR20, which eventually resulted in enhanced hardness or reduced hardness in those specimens, respectively. Figure 11a illustrates the schematic of the atomic cluster in an as-cast glassy phase, which is modified as a result of the alteration of the free volume content and subsequent dilation or densification at the later stage of cold rolling, as shown in Figure 11b,c, respectively. The relaxed glassy phase in CR4.5 and CR31 exhibited densification-induced hardening during nanoindentation. However, such deformation-induced densification was also reported earlier in the case of silicate and polymeric glasses [55,56,57]. Cold rolling induced structural relaxation, accompanied by reduced free volume content, as reported by us earlier [34]. Thus, the free volume accumulation and annihilation play crucial roles during cold rolling in Vitreloy 1.

## 5. Conclusions

The following conclusions were drawn from the present study:

- The hardness at the specimen surface varied with cold rolling percentage (%) and the variation is similar on the RW and NR planes at all different peak loads in the range of 50 mN–500 mN, whereas the same is insignificant for the core region of the specimen on the NW plane. *H* and *E* values increase in CR4.5 and CR31, whereas CR10 and CR20 become softer than CR0 upon cold rolling on the RW and NR planes.
- 3D optical surface profilometry studies on the NR plane suggest that the shear band spacing decreases from 57.7 μm (CR4.5) to 24.6 μm (CR31), and the shear band offset height increases from 2.4 μm (CR4.5) to 4.4 μm (CR31) with the increase in the extent of cold rolling. Meanwhile, the number of pop-in events during nanoindentation for all the planes reduces with the increase in the extent of cold rolling, and the disappearance of shear bands around indentation impression points to more homogeneous deformation in cold rolled BMGs.
- The nanoindentation, DSC studies, and model calculations suggest that the annihilation rate dominated over the free volume creation rate, which produce a relaxed and dense glassy structure in CR4.5 and CR31, exhibiting enhanced *H* and *E*.

## Figures and Tables

**Table 1:** *H* and *E* values of the NR plane in CR0, CR4.5, CR10, CR20, and CR31 at different *P*_max_ values in between 50 and 500 mN.

	*H* (GPa) NR				*E* (GPa) NR			
*P*_Max_ (mN)	50 mN	100 mN	200 mN	500 mN	50 mN	100 mN	200 mN	500 mN
CR0	18.9 ± 0.6	15.9 ± 0.1	12.6 ± 0.9	9.5 ± 0.1	187 ± 5	163 ± 3	140 ± 1	117 ± 1
CR4.5	31.0 ± 0.6	26.3 ± 2.0	19.6 ± 0.4	14.7 ± 0.9	241 ± 4	189 ± 4	159 ± 2	121 ± 1
CR10	16.9 ± 0.8	13.9 ± 0.5	10.9 ± 0.5	9.0 ± 0.1	169 ± 2	144 ± 2	122 ± 1	102 ± 2
CR20	18.0 ± 0.3	15.3 ± 0.4	12.2 ± 0.6	9.5 ± 0.1	175 ± 3	154 ± 1	133 ± 2	107 ± 2
CR31	32.6 ± 1.6	27.3 ± 0.6	20.3 ± 0.9	16.4 ± 1.2	291 ± 12	248 ± 4	204 ± 4	167 ± 6

**Table 2:** *H* and *E* values of the RW plane in the as-cast and differently cold rolled samples at different maximum loads of 50 mN, 100 mN, 200 mN, and 500 mN.

	*H* (GPa) RW				*E* (GPa) RW			
*P*_Max_ (mN)	50 mN	100 mN	200 mN	500 mN	50 mN	100 mN	200 mN	500 mN
CR0	21.9 ± 0.2	16.7 ± 3.3	13.8 ± 0.1	9.6 ± 2	204 ± 4	153 ± 3	135 ± 1.5	109 ± 8
CR4.5	32.1 ± 3.3	20.5 ± 0.8	17.5 ± 2.1	11.8 ± 0.5	244 ± 11	175 ± 2	153 ± 8	110 ± 2
CR10	16.5 ± 0.2	13.2 ± 0.3	9.9 ± 0.1	8.0 ± 0.2	161 ± 2	141 ± 3	111 ± 1	90 ± 0.3
CR20	20.3 ± 1.6	16.0 ± 1.8	13.6 ± 0.1	8.9 ± 0.6	186 ± 5	161 ± 6	141 ± 1	106 ± 3
CR31	26.8 ± 3.0	21.7 ± 2.1	18.2 ± 1.3	13.9 ± 0.7	246 ± 12	181 ± 3	158 ± 6	112 ± 1

**Table 3:** *H* and *E* values of the NW plane in CR0, CR4.5, CR10, CR20, and CR31 at various *P*_max_ values in between 50 and 500 mN.

	*H* (GPa) NW				*E* (GPa) NW			
*P*_Max_ (mN)	50 mN	100 mN	200 mN	500 mN	50 mN	100 mN	200 mN	500 mN
CR0	7.9 ± 0.3	7.4 ± 0.3	6.8 ± 0.1	5.9 ± 0.2	104 ± 2	97 ± 2	87 ± 1	73 ± 1
CR4.5	7.6 ± 0.7	6.8 ± 0.1	6.2 ± 0.2	5.7 ± 0.1	100 ± 4	90 ± 0.2	74 ± 2	58 ± 0.4
CR10	7.9 ± 0.3	7.1 ± 0.4	6.2 ± 0.1	5.5 ± 0.1	111 ± 2	108 ± 3	102 ± 2	94 ± 2
CR20	8.4 ± 0.5	7.7 ± 0.5	6.9 ± 0.3	6.3 ± 0.3	109 ± 3	96 ± 2	82 ± 2	68 ± 2
CR31	7.9 ± 0.3	6.9 ± 0.3	6.3 ± 0.3	5.5 ± 0.1	94 ± 3	84 ± 2	74 ± 1	57 ± 0.5

**Table 4:** The affected deformed volume in CR specimens during nanoindentation on the NR, RW, and NW planes at different *P*_max_.

	Affected Volume (µm)^3^											
	NR				RW				NW			
*P*_max_ (mN)	50	100	200	500	50	100	200	500	50	100	200	500
CR0	0.6^3^	1.0^3^	1.8^3^	3.6^3^	0.5^3^	0.9^3^	1.7^3^	3.3^3^	1.2^3^	1.8^3^	2.6^3^	4.3^3^
CR4.5	0.4^3^	0.8^3^	1.3^3^	2.6^3^	0.4^3^	0.8^3^	1.4^3^	3.1^3^	1.2^3^	1.7^3^	2.6^3^	4.4^3^
CR10	0.6^3^	1.1^3^	2.0^3^	3.6^3^	0.6^3^	1.2^3^	2.1^3^	3.8^3^	1.2^3^	1.8^3^	2.7^3^	4.5^3^
CR20	0.6^3^	1.1^3^	1.8^3^	3.5^3^	0.5^3^	1.0^3^	1.7^3^	3.7^3^	1.2^3^	1.8^3^	2.7^3^	4.2^3^
CR31	0.4^3^	0.7^3^	1.3^3^	2.6^3^	0.4^3^	0.8^3^	1.4^3^	2.8^3^	1.3^3^	1.8^3^	2.6^3^	4.3^3^

## Data Availability

The data presented in this study are available in this article.

## Supplementary Materials

The following are available online at https://www.mdpi.com/article/10.3390/nano11071670/s1, Figure S1: *P-h* plots at *P*_max_ = 200 mN along along NR, RW and NW; Figure S2: *P-h* plots at *P*_max_ = 100 mN along along NR, RW and NW; Figure S3: *P-h* plots at *P*_max_ = 50 mN along NR, RW and NW.
