# Supplementary material for: Effect of Cold Rolling on the Evolution of Shear Bands and Nanoindentation Hardness in Zr41.2Ti13.8Cu12.5Ni10Be22.5 Bulk Metallic Glass
Source: Nanomaterials (Basel). 2021 Jun 25;11(7):1670. doi: 10.3390/nano11071670 (PMC8307797; doi:10.3390/nano11071670)
Supplement: Supplementary file 1 [file nanomaterials-11-01670-s001.zip › nanomaterials-1204789-supplementary.pdf]

Supplementary information

Effect of Cold Rolling on the Evolution of Shear Bands and Nanoindentation Hardness in $Zr_{41.2}Ti_{13.8}Cu_{12.5}Ni_{10}Be_{22.5}$ Bulk Metallic Glass

Abhilash Gunti [1], Parijat Pallab Jana [1], Min-Ha Lee [2], and Jayanta Das [1,*]

[1] Department of Metallurgical and Materials Engineering, Indian Institute of Technology Kharagpur, West Bengal 721302, India; abhilash@iitkgp.ac.in (A.G.); parijat.pallab@iitkgp.ac.in (P.P.J.)

[2] KITECH North America, Korea Institute of Industrial Technology, San Jose, CA 95134, USA; mhlee1@kitech.re.kr

* Correspondence: j.das@metal.iitkgp.ac.in; Tel.: +91-3222-283284; Fax: +91-3222-282280

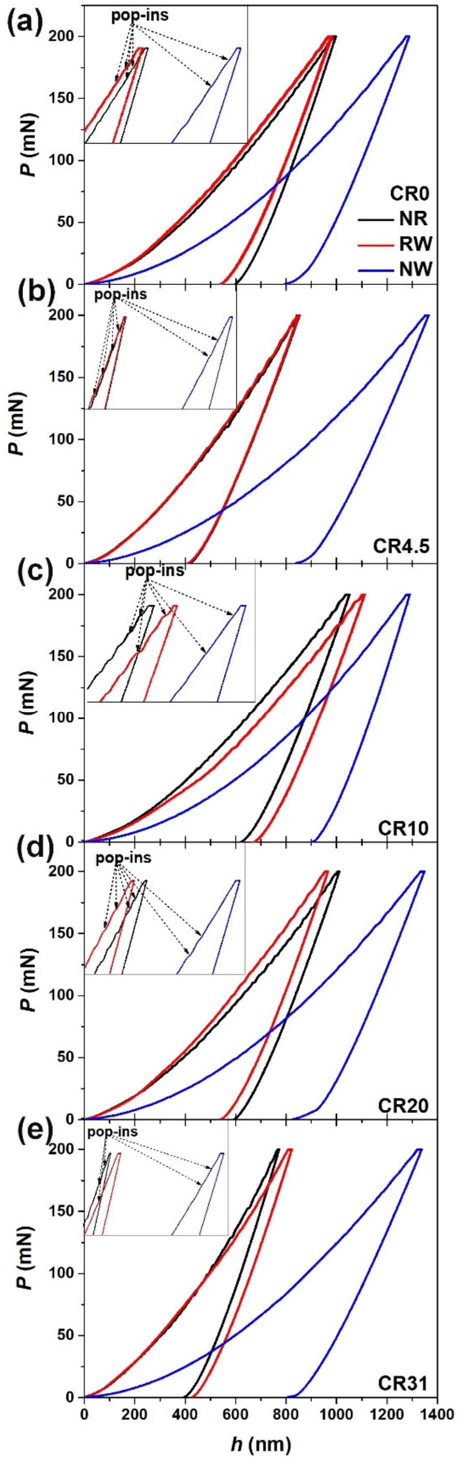

Figure S1: *P*- *h* plots at $P_{\max}$ = 200 mN along along NR, RW and NW; (**a**) CR0, (**b**) CR4.5, (**c**) CR10, (**d**) CR20 and (**e**) CR31, pop-in events are more pronounced in CR0, which gradually decreases with the increase of cold rolling strain.

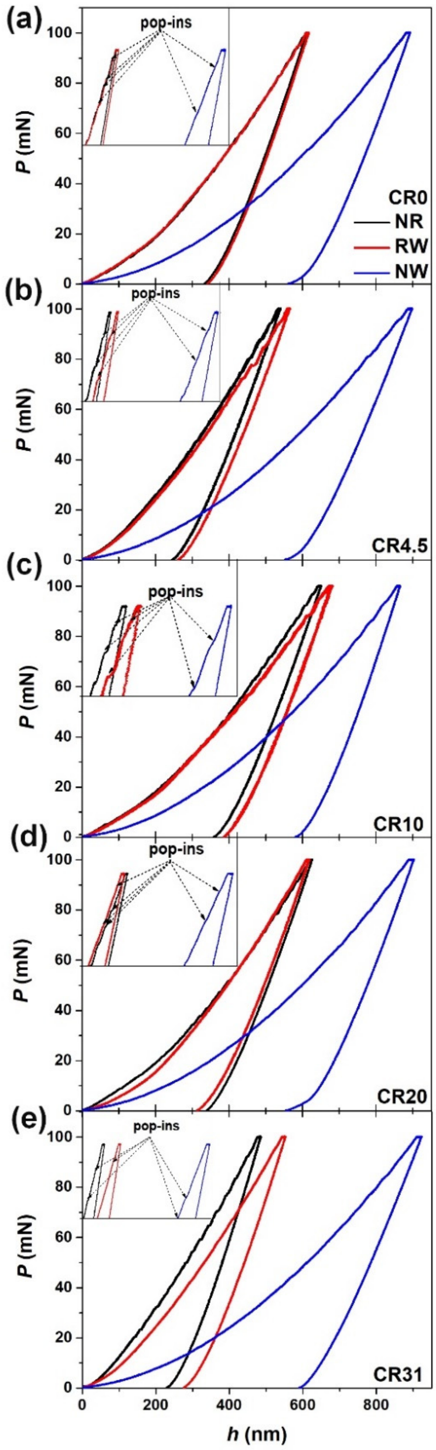

Figure S2: *P*- *h* plots at P_{max} = 100 mN along along NR, RW and NW; (**a**) CR0, (**b**) CR4.5, (**c**) CR10, (**d**) CR20 and (**e**) CR31, pop-in events are more pronounced in CR0, which gradually decreases with the increase of cold rolling strain.

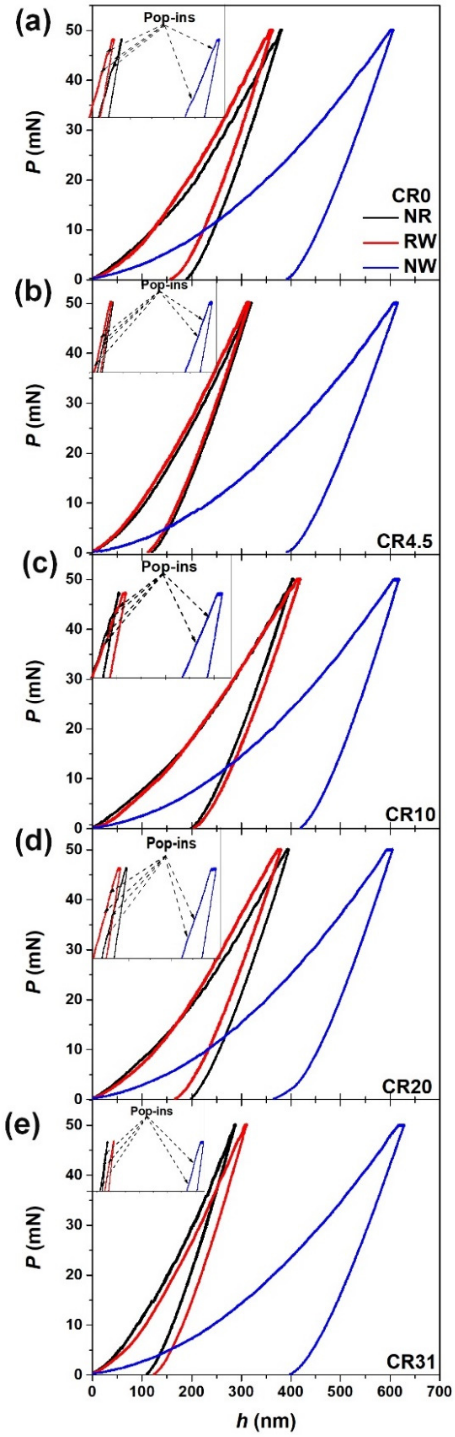

Figure S3: *P*- *h* plots at $P_{\max}$ = 50 mN along NR, RW and NW; (**a**) CR0, (**b**) CR4.5, (**c**) CR10, (**d**) CR20 and (**e**) CR31, pop-in events are more pronounced in CR0, which gradually decreases with the increase of cold rolling strain.
